# Arsenic Exposure and Risk of Urothelial Cancer: Systematic Review and Meta-Analysis

**DOI:** 10.3390/ijerph17093105

**Published:** 2020-04-29

**Authors:** Pamela Di Giovanni, Giuseppe Di Martino, Piera Scampoli, Fabrizio Cedrone, Francesca Meo, Giuseppe Lucisano, Ferdinando Romano, Tommaso Staniscia

**Affiliations:** 1 Department of Pharmacy, “G. d’Annunzio” University Chieti-Pescara, Via dei Vestini 31, 66100 Chieti, Italy; pamela.digiovanni@unich.it; 2 Department of Medicine and Aging Sciences, “G. d’Annunzio” University Chieti-Pescara, Via dei Vestini 31, 66100 Chieti, Italy; tommaso.staniscia@unich.it; 3 School of Hygiene and Preventive Medicine, “G. d’Annunzio” University Chieti-Pescara, Via dei Vestini 31, 66100 Chieti, Italy; piera.scampoli@gmail.com (P.S.); cedronefab@gmail.com (F.C.); francesca.meo10@gmail.com (F.M.); 4 Centre for Outcomes Research and Clinical Epidemiology (CORESEARCH), Via Tiziano Veciello, 65100 Pescara, Italy; lucisano@coresearch.it; 5 Department of Public Health and Infectious Diseases, “La Sapienza” University of Rome, P.zza Aldo Moro 5, 00100 Rome, Italy; ferdinando.romano@uniroma1.it

**Keywords:** arsenic, urothelial cancer, meta-analysis, drinking water

## Abstract

*Background*: Arsenic is a toxic metalloid element widely distributed throughout the environment. Arsenic contaminated water has become an ongoing public health issue affecting hundred million people worldwide. The aim of this paper was to summarize the evidence in the association between arsenic metabolites and urinary tract cancer risk. *Methods*: A systematic review was conducted searching for observational studies that evaluated the association of arsenic metabolites and urinary tract cancer. Risk estimates from individual studies were pooled by using random effects models. *Results*: All the metabolites considered in this study resulted to be significantly associated to urothelial cancer, respectively: IA% 3.51 (1.21–5.82) (*p* = 0.003), MMA with WMD = 2.77 (1.67–3.87) (*p* < 0.001) and DMA with WMD = −4.56 (−7.91–1.22) (*p* = 0.008). *Conclusions*: Arsenic metabolites are significantly associated to urothelial cancer. Future studies will help to verify the independent association(s) between arsenic metabolites and urothelial cancer.

## 1. Introduction

Arsenic (As) is a toxic metalloid element widely distributed throughout the environment. It can be found in rocks, soil, water, air, or in plants and animals in organic or inorganic forms. Naturally high levels of arsenic are present in the groundwater of a number of countries (including Argentina, Bangladesh, Chile, China, India, Mexico and the USA) [1,2]. Arsenic may also be released into the environment through natural activities such a rock erosion, or human activities such as farming and industry [1,2,3]. Exposure to arsenic from drinking water is the primary route among highly exposed populations [4,5,6,7]. Ingested As is eliminated in the urine as monomethylated metabolites (MMAs), in a lower proportion than dimethylated ones (DMAs), as well as inorganic As (IA) [8]. Arsenic- contaminated water has become an ongoing public health issue affecting hundreds of millions of people worldwide. Furthermore, evidence supporting a wide range of acute and chronic health effects, including cancer, has led the World Health Organization (WHO) to lower the advisory limit for As concentration in drinking water from 25 μg/L to 10 μg/L [9]. The International Agency for Research on Cancer (IARC) has classified inorganic As in drinking water as a Group 1 carcinogen [10]. The carcinogenic effects of As include oxidative damage, epigenetic effects, and interference with DNA repair, all implicated in the development of urinary tract cancer [11,12]. To this extent, several studies have been carried out to investigate the association between As and urinary tract cancer in different populations. 

Review Question

The aim of this paper was to summarize the evidence in the association between As metabolites and urinary tract cancer risk provided by up-to-date research outcomes.

## 2. Materials and Methods 

### 2.1. Outcomes

The primary outcomes evaluated were:

- Mean Difference (MD) (cases vs. controls) of IA (%);
- Mean Difference (MD) (cases vs. controls) of DMA (%);
- Mean Difference (MD) (cases vs. controls) of MMA (%).

A sensitivity analysis was conducted on odds ratios reported in the included papers about the risk of urothelial cancer of cases vs. controls. In this case, the exposure of IA was classified as high or low (not continuous). The cutoffs to define as high/low IA% changed between the studies. 

### 2.2. Search Strategy and Study Selection

The systematic review was conducted according to the “Conducting Systematic Reviews and MetaAnalyses of Observational Studies of Etiology” (COSMOS-E) guidelines [13]. Only observational studies publicly available on 14 March 2020 and reporting on exposure to As in drinking water and urinary tract cancer were identified. PubMed/Medline, Web of Sciences (WoS), Science Direct and Google Scholar were searched. The research strategy was reported as Supplementary Material (SM1). The inclusion criteria were the following: studies involving human participants, original research paper evaluating the association among arsenic metabolites and urinary tract cancer, original research paper that reported almost one arsenic metabolite dosage. The exclusion criteria were the following: studies involving non-human populations, publications containing non-original research (i.e., reviews, editorials, letters to editor), case reports and case series, studies that did not measure urinary tract cancer, studies that did not report information on arsenic metabolism, ecological studies and studies that did not report dosages of arsenic metabolites. In addition, non-English papers were also excluded. 

### 2.3. Data Extraction

Two authors, P. Scampoli and F. Meo, independently screened titles and abstracts of retrieved citation in order to identify potentially eligible studies. Each discrepancy was discussed with a third investigator, T. Staniscia. All potentially relevant citations were then retrieved in full-text and independently reviewed by two investigators, F. Cedrone and G. Di Martino Data meeting the selection criteria were independently extracted from articles by two investigators, G. Di Martino and P. Scampoli, and reported in a specific digital form. The variables considered for this systematic review were: study name, year, country, study design, number of participants, mean of IA%, MMA% and DMA% among cases and controls, risk estimates, factors considered in the adjusted analysis and conclusions. All the extracted data were checked by two investigators, P. Di Giovanni and F. Romano. Two investigators (F. Cedrone and F. Meo) assessed the study quality according to the Newcastle-Ottawa Quality Control Scale [14].

### 2.4. Statistical Analysis

The characteristics of included studies were showed in Table 1. Weighted Mean Differences (WMD) with 95% confidence intervals (95%CI) were defined according to Cases minus Controls. Secondly, we pooled risk estimates from individual studies by using random effects models [15] for the sensitivity analysis. We used the heterogeneity χ2 (Cochran Q) statistic to formally analyze heterogeneity across included studies [16]. When Cochran Q *p*-values was < 0.10, we pooled risk estimates using random effects models. The heterogeneity was also represented by I2 index. The potential for publication bias was assessed using a funnel plot in conjunction with Kendall’s Tau, unweighted and weighted Weighted Egger Intercept, Macskill’s inverse variance weighted and Macskill’s inverse pooled variance weighting. All analyses were carried out using R version 3.2.0.3.

## 3. Results

### 3.1. Studies Selection

**Figure 1:**
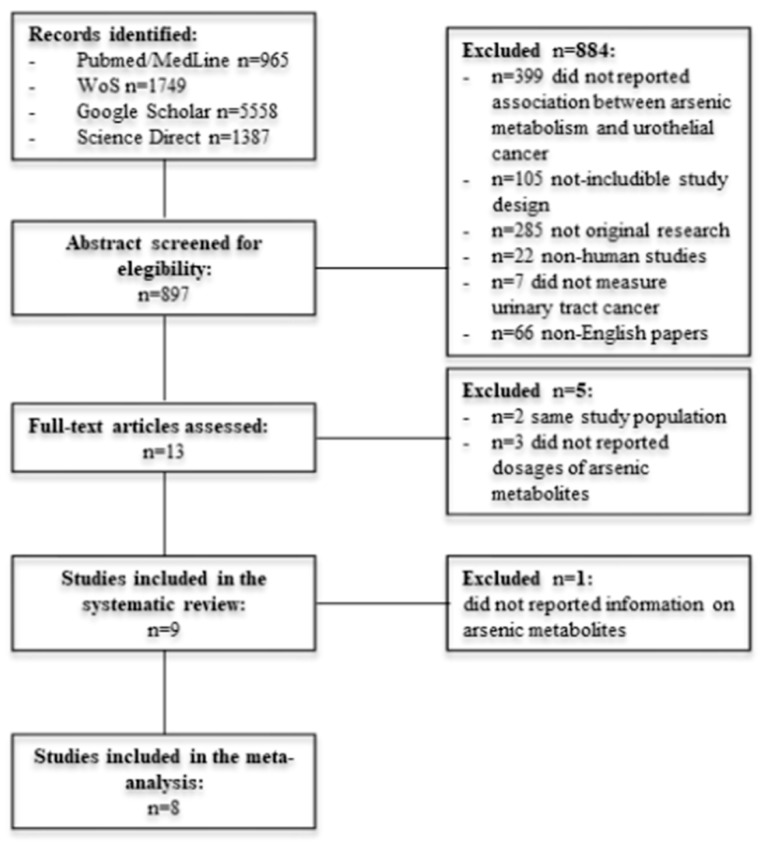
Flowchart of the study selection.

Electronic searching through 14 March 2020 retrieved 9659 articles, as shown in Figure 1. From the 9659, 8762 were excluded after title screening. Of the remaining 897 papers, 884 were excluded after abstract screening: 399 did not report and association between arsenic metabolism and urothelial cancer, 285 were not original researches, 22 involved non-human subjects or were in-vitro studies, 105 were performed with a study design that did not match the inclusion criteria, seven did not measure urinary tract cancer and 66 were non-English language papers. Of 14 papers, only nine met inclusion criteria and were included in the systematic review after full-text screening [17,18,19,20,21,22,23,24,25]. In particular, two papers reported on the same study population as other included papers, and three did not report the dosages of arsenic metabolites. Eight studies were thus ultimately included in the meta-analysis. One paper was excluded from the meta-analysis because it only reported the risk estimates of urothelial cancer and did not report metabolite dosages in cases and in non-cases. 

### 3.2. Characteristics of Included Studies

**Figure 2:**
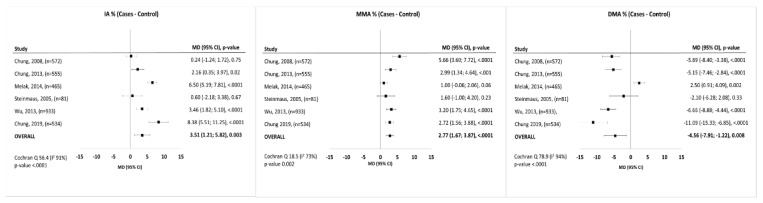
Forest-plot of weighted mean differences in arsenic metabolites between cases and control.

Characteristics of included studies are shown in Table 1. Most study designs were case-controls (eight studies), and were conducted in Taiwan (seven studies). All studies were performed between 2007 and 2019. The number of participants was heterogeneous, ranging from 81 [23] to 933 [20] for case-control studies. The cohort study by Chung et al. [17] enrolled 28 participants. The studies from Chung et al. [17] and Huang et al. [22], did not reported mean values of arsenic metabolites in cases and controls. The studies by Chung et al. [25] and Steinmaus et al. [23] reported the highest mean concentrations of IA and MMA. In Chung et al. they were 15.1% and 12.4% respectively, while in Steinmaus et al. 13.3%, and 13.7% respectively. The lower concentration of IA and MMA were reported by Pu et al. [21], respectively 5.9% and 9.9%. Most studies showed significantly higher mean difference of either IA [19,20,24,25] or MMA [18,20,24,25] among both cases and controls. DMA concentration was significantly lower in cases in four studies [17,18,20,25], whereas only one study reported opposite/divergent results [19]. Four studies analyzed metabolites concentration as tertiles [17,20,21,25] and one as quartile [22]. Only one study dichotomized these variables by median values [18]. Melak et al. [18] reported only risk estimate for MMA%. Chung et al. [25] analyzed also the risk of UC by tertiles of distribution, without showing the values of every tertiles. Most of the included studies adjusted risk estimates at least for age and gender [17,18,19,20,21,22,24,25]. Four studies adjusted risk estimates also for smoking status [19,22,24,25], three for alcohol consumption [20,21,22] and five for educational level [20,21,22,24,25]. Two studies [21,25] adjusted the analysis also for pesticides exposure and only one study adjusted also for parents ethnicity [21]. As reported in Figure 2, six studies concluded that IA was significantly associated with UC [17,18,20,22,24,25], nine studies reported that MMA was significantly associated with UC [17,18,19,20,21,22,23,24,25], and seven studies reported that DMA was significantly associated with UC [17,18,20,21,22,24,25]. Among included studies, four studies [18,20,21,22] were performed at Taiwan University Hospital in the same period, so the possibility of patients overlap cannot be excluded.

### 3.3. Meta-Analysis

**Figure 3:**
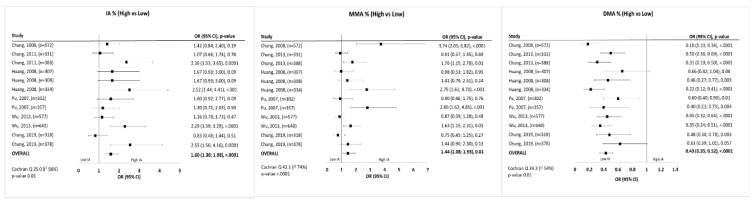
Forest-plot of association between arsenic metabolites distribution and urothelial cancer.

The first analysis was focused on the summary association between UC and As metabolites. It considered metabolites as continuous variables and included a total of 3,140 patients taken into account for six studies (Figure 2). All the metabolites considered in this study were significantly associated to urothelial cancer, respectively: IA% with WMD = 3.51 (1.21–5.82) (*p* = 0.003), MMA with WMD = 2.77 (1.67–3.87) (*p* < 0.001) and DMA with WMD = −4.56 (−7.91–1.22) (*p* = 0.008) as shown in Figure 2. Sensitivity analysis considered a total of 3539 patients and summarized the risk of higher concentration of As metabolites on urothelial cancer. It showed that both MMA, DMA and IA were significantly associated with urothelial cancer (Figure 3). Particularly, a higher concentration of IA had an overall OR of 1.60 (1.30–1.98; *p* < 0.001), a higher concentration of MMA had overall OR of 1.44 (1.08–1.93; *p* = 0.010), and a higher concentration of DMA had overall OR of 0.43 (0.35–0.52; *p* < 0.001). The study by Chung et al. [25] also presented the risk estimates by tertiles of distribution, without reporting the exact value of each tertile, so it was considered only in the first analysis. The Cochran Q test *p*-values was <0.10 for all outcomes and for this reasons we pooled risk estimates using random effects models. Every publication bias method suggests the absence of publication bias (Tables S2 and S3). Even the funnel plots showed a symmetrical distribution of the study estimates (Figures S1 and S2).

### 3.4. Study Design and Quality Assessment

Eight studies included in the systematic review were case-control [18,19,20,21,22,23,24,25], and one was a prospective cohort study [17]. All of them showed marked heterogeneity in reporting and modelling biomarkers of arsenic metabolism (Table S1). Among case control studies, most of them did not describe the case control selection and the definition of controls.

## 4. Discussion

### 4.1. Main Findings

This systematic review on arsenic metabolism and UC gives evidence to both the associations between UC and higher IA and between higher MMA and lower DMA, respectively. Specifically, the mean concentration difference between cases and controls was significant for IA, MMA, and DMA. All the metabolites kept a significant association also when patients presenting high metabolites concentration were compared with patients presenting lower metabolites concentration. The metabolites concentration level was quite different across the studies and ranged between 4.53 and 15.1. This was particularly the case of IA. Conversely, MMA and DMA reported lower variability. These differences in human populations was consistent with several studies on animals [26]. According to this meta-analysis, there is an increase of over 50% in UC risk when the extreme MMA categories are compared. This is consistent with the evidence showing that MMA is a genotoxic metabolite that directly cuts supercoiled DNA [27] and causes chromosomal breaks in lymphocyte cultures through reactive oxygen species generation [28].

Additionally, MMA subtypes decrease DNA repair ability by affecting the cleavage of 8-oxoguanine adducts, as observed afterwards in vitro experiments employing lung cells. Furthermore, there is evidence that MMA might cause cancer through histone modifications as epigenetic mechanism, as observed in the UROtsa cell line [29]. In this meta-analysis, the significant association of DMA proved to have a protective effect on UC. Nevertheless, this result should be interpreted by the knowledge of As metabolism: in particular DMA is complementary to MMA, so their risk estimates are opposite. Some experimental evidence showed as also DMA had carcinogenic effect on human [27,30]. These findings are in line with previous meta-analyses [31,32]. Moreover, arsenic or its metabolites may decrease DNA methylation and may be involved in tumor formation and progression [25].

Human exposure to As is a major public health concern. People can be exposed to As in different ways such as drinking water, food, and air pollution. In addition, many risk factors could enhance the risk of UC in people exposed to As, such as smoking, low folate intake and pesticides exposure [18,33]. In particular, smoking habits does not influence total As urinary levels, but probably interferes with arsenic methylation capacity [25]. In addition, smoking is an independent known risk factor for UC [34]. For these reasons, all included studies adjusted the analysis for smoking habits. Also alcohol consumption is an independent risk factor for UC [35], but only three included studies [20,21,22] were adjusted for it. 

No included studies reported the dose-response relationship among UC and As metabolites. This is due to the study design that cannot allow evaluating both the prospective As metabolites urinary levels and both the exposure dose of involved patients in drinking water. A recent meta-analyses tried to summarize evidence of dose effect of As concentration in drinking water on UC risk, showing lack of an increased risk of bladder cancer in categories of exposure with mean/midpoint up to 150 mg/L. All studies included in our meta-analysis reported urinary concentration of As metabolites, and not As level in drinking water, so the results of these two meta-analyses cannot be overlapped and compared.

### 4.2. Strengths and Limitations

The strength of this study was the analysis of As metabolites both as continuous variables and as categorical variables. The main findings were confirmed by both types of analysis that gave particular strength to our study. Despite this, the results should be interpreted in the light of some methodological limitations. First the heterogeneity in the description and analysis of arsenic metabolism. Modelling methods performed across the studies were adjusted for different factors, so the possibility of residual confounding cannot be excluded. Second, despite these results, the possibility of publication bias cannot be ignored, as null associations may be less likely to be published. Publication bias may overestimate the consistency of the relationship between arsenic metabolism and UC. In particular, cohort studies with no cases of UC and studies with non-significant association among UC and arsenic metabolites were not found in our research. In addition, as all the analysed studies were case–controlled ones, the possibility of reverse causation cannot be excluded. In fact, in case–control studies UC is a prevalent case and the collection of blood and urine samples was performed after the diagnosis of cancer. So, these measurements may be considered reliable only if we assume that the exposure to As and its metabolism remain stable in time. Also, some studies were performed in the same hospital, so the possibility of patients overlap among studies cannot be excluded due to anonymous data reported in all studies. Finally, the great part of included studies (six of nine) was performed in the same country (Taiwan), and it can make these results less generalizable. From included studies is not possible to find a threshold for the carcinogenic risk of As metabolites. So public health decision-makers need focused prospective studies both to evaluate the minimum level of risky As metabolism concentrations, both to evaluate the risk of long time exposure to As. However, the consistency of these findings sets the stage for additional research to determine the causality of these associations. 

## 5. Conclusions

Arsenic metabolites levels in urine resulted significantly associated with urothelial cancer. The presence of As in soil and drinking water is a public health concern due to its possible role in urinary tract carcinogenesis. Public health policies and intervention should be aimed to improve surveillance and preventive strategies and to protect population exposed to arsenic. Future studies using large sample size, appropriate baseline, and prospective arsenic metabolism estimation will help to verify the independent association between arsenic metabolites and UC. In addition, a prospective evaluation of chronic exposure and As metabolism over time, could help in assessing the dose-response effect on urinary tract and its impact on UC development. 

## Figures and Tables

**Table 1:** Characteristics of included studies.

Study	Year	Country	Study Design	Participants	IA% Cases (Mean)	MMA% Cases (Mean)	DMA% Cases (Mean)	Risk Estimate Contrast IA%	Risk Estimate IA%	Risk Estimate Contrast MMA%	Risk Estimate MMA%	Risk Estimate Contrast DMA%	Risk Estimate DMA%	Adjustment	Conclusion
Chung	2008	Taiwan	Case-Control	572	7.18	13.19	79.63	>4.32 vs. <4.32	1.42	>6.1 vs. <6.1	3.74	>88 vs. <88	0.18	Age and gender	MMA and DMA were associated with UC
Chung	2013	Taiwan	Case-Control	555	9.06	10.53	80.41	2.76–5.86 vs. <2.76	1.07	3.36–9.13 vs. <3.36	0.91	>91.76 vs. 83.56–91.76	0.5	Age and gender	IA, MMA and DMA were associated with UC
								>5.86 vs. <2.76	2.36	>9.13 vs. <3.36	1.76	>91.76 vs. <86.56	0.31		
Chung1	2013	Taiwan	Cohort	28				4.22–7.87 vs. <4.22	2.42	8.34–15.31 vs. <8.34	0.57	>85.8 vs. 76.13–85.8	1.43	Age, gender, education and smoking habits	IA, MMA and DMA were associated with UC
								>7.86 vs. <4.22	3.53	>15.32 vs. <8.34	1.77	>85.8 vs. >76.13	0.33		
Huang	2008	Taiwan	Case-Control	659				1.5–3.69 vs<1.49	1.67	0.9–5.89 vs. <0.89	0.98	81.9–89.19 vs. <81.89	0.66	Age, gender, educational attainment, smoking status, and alcohol consumption	IA, MMA and DMA were associated with UC
								3.70–6.29 vs. <1.49	1.67	5.9–10.89 vs. <0.89	1.41	89.20–94.39 vs. <81.89	0.46		
								>6.30 vs. <1.49	2.52	>10.90 vs. <0.89	2.75	>94.40 vs. <81.89	0.22		
Pu	2007	Taiwan	Case-Control	490	5.9	9.9	84.2	2.5–5.2 vs. <2.4	1.6	3.1–9.2 vs. <3	0.9	85.1–92.5 vs. <85	0.6	Age, gender, education, parents ethnicity, alcohol and pesticides exposure	IA, MMA and DMA were associated with UC
								> 5.3 vs. <2.4	1.2	>9.3 vs. <3	2.8	>92.6 vs. <85	0.4		
Melak	2014	Chile	Case-Control	464	10.4	11.2	81.9			>12.5 vs. <12.5	1.41			Age, gender, and smoking	MMA was associated with UC
Steinmaus	2005	USA	Case-Control	81	13.3	13.7	73							Age, gender, education, pesticide exposure and smoking habits	MMA increased UC risk
Wu	2013	Taiwan	Case-Control	933	9.71	10.55	79.74	2.61–5.7 vs. <2.61	1.16	3.04–8.87 vs. <3.04	0.87	84.72–92.50 vs. <84.72	0.45	Age, gender, education and alcohol consumption	IA, MMA and DMA were associate with UC
								>5.8 vs. <2.61	2.29	>8.88 vs. <3.04	1.63	>92.51 vs. <84.72	0.35		
Chung	2019	Taiwan	Case-Control	534	15.1	12.47	73.33	Q1 vs. Q2 Q1 vs. Q3	0.83 2.55	Q1 vs. Q2 Q1 vs. Q3	0.75 1.44	Q1 vs. Q2 Q1 vs. Q3	0.48 0.63	Age, gender, education, pesticides exposure and smoking habits	IA, MMA and DMA were associated with UC

## Supplementary Materials

The following are available online at https://www.mdpi.com/1660-4601/17/9/3105/s1, Research strategies; Table S1. Quality assessment according to Newcastle-Ottawa Quality Control Scale; Table S2. Assessment of publication bias considering metabolites as continuous variables; Table S3. Assessment of publication bias considering metabolites as dichotomous variables; Figure S1. Funnel plots performed to evaluate publication bias for metabolites reported as continuous variables; Figure S2. Funnel plots performed to evaluate publication bias for metabolites reported as categorical variables.
