# Supplementary material for: Arsenic Exposure and Risk of Urothelial Cancer: Systematic Review and Meta-Analysis
Source: Int J Environ Res Public Health. 2020 Apr 29;17(9):3105. doi: 10.3390/ijerph17093105 (PMC7246722; doi:10.3390/ijerph17093105)

Supplementary material

SM1. Research strategies:

Pubmed: ((arsenic[MesH Term]) OR (arsenic)) AND ((urothelial) OR (bladder) OR (kidney)) AND ((cancer) OR (tumor) OR (neoplasia) OR (neoplasm)) = 965

WoS: (Arsenic) AND ((urothelial) OR (bladder) OR (kidney)) AND ((cancer) OR (tumor) OR (neoplasia) OR (neoplasm)) = 1749

Scholar: (Arsenic) AND (metabolites) AND ((urothelial) OR (bladder) OR (kidney)) AND ((cancer) OR (tumor) OR (neoplasia) OR (neoplasm)) = 5558

Sciences Direct: (Arsenic) AND (metabolites) AND ((urothelial) OR (bladder) OR (kidney)) AND ((cancer) OR (tumor) OR (neoplasia) OR (neoplasm)) = 1387

Table S1. Quality assessment according to Newcastle-Ottawa Quality Control Scale

Study	Case Definition	Representativeness of the Cases	Selection of Controls	Definition of Controls	Comparability of Cases and Controls	Ascertainment of Exposure	Non-Response Rate
**Chung 2008**	◊	◊		◊	◊◊	◊	◊
**Chung 2013**	◊	◊	◊		◊◊	◊	◊
**Huang 2008**	◊	◊		◊	◊◊	◊	◊
**Melak 2014**	◊		◊		◊◊	◊	◊
**Pu 2007**	◊	◊		◊	◊◊	◊	◊
**Steinmaus 2005**	◊	◊	◊			◊	◊
**Wu 2013**	◊	◊		◊	◊◊	◊	◊
**Chung 2019**	◊	◊	◊	◊	◊◊	◊	◊

Table S2. Assessment of publication bias considering metabolites as continuous variables

Test	IA%	DMA	MMA
	p-value	p-value	p-value
Begg's Method (Kendall's Tau)	0.88193	0.41318	0.41318
Unweighted Egger Intercept	0.93436	0.15411	0.28217
Weighted Egger Intercept	0.59340	0.05185	0.14350
Macskill's Inverse Variance Weighting	0.96406	0.28559	0.38110
Macskill's Inverse Pooled Variance Weighting	0.83126	0.57576	0.69834

Table S3. Assessment of publication bias considering metabolites as dichotomous variables

Test	IA% p-value	DMA p-value	MMA p-value
Begg's Method (Kendall's Tau)	0.71938	0.00421	0.62158
Unweighted Egger Intercept	0.71352	0.12703	0.64436
Weighted Egger Intercept	0.66279	0.31680	0.69700
Macskill's Inverse Variance Weighting	.	.	.
Macskill's Inverse Pooled Variance Weighting	.	.	.

Figure S1. Funnel plots performed to evaluate publication bias for metabolites reported as continuous variables

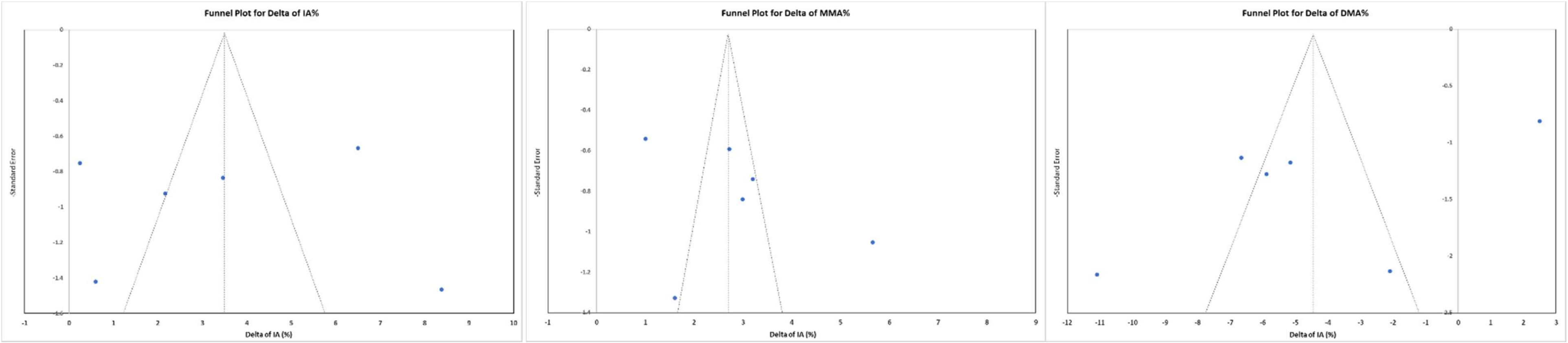

Figure S2. Funnel plots performed to evaluate publication bias for metabolites reported as categorical variables

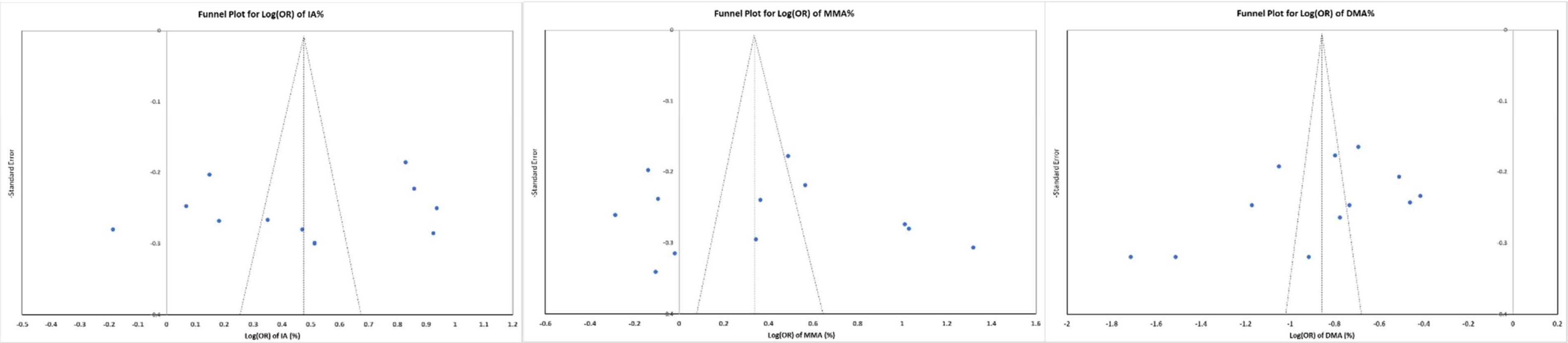

Supplement: Supplementary file 1 [file ijerph-17-03105-s001.zip › ijerph-729365-supplementary.pdf]
